# Delayed Traumatic Subarachnoid Hemorrhage Following Initially Negative Computed Tomography (CT) Imaging in a Patient With a Mechanical Mitral Valve and Markedly Elevated International Normalized Ratio (INR)

**DOI:** 10.7759/cureus.113580

**Published:** 2026-07-29

**Authors:** Kelsey N Sauers, Paris L Tomlinson, Talyr E Hall

**Affiliations:** 1 Internal Medicine/Pediatrics, Edward Via College of Osteopathic Medicine, Auburn, USA; 2 Neurology, Edward Via College of Osteopathic Medicine, Auburn, USA; 3 Internal Medicine, Hospital Corporation of America (HCA) Florida Fort Walton-Destin Hospital, Fort Walton Beach, USA

**Keywords:** anticoagulation, drug-drug interaction, heparin bridging, intracranial hemorrhage, mechanical heart valve, repeat imaging, subarachnoid hemorrhage, supratherapeutic inr, vitamin k, warfarin

## Abstract

Warfarin remains the anticoagulant of choice for patients with mechanical heart valves because of its effectiveness in preventing thromboembolic events. However, management becomes particularly challenging when severe supratherapeutic anticoagulation is complicated by intracranial hemorrhage. We present the case of a 76-year-old male patient with a mechanical mitral valve on chronic warfarin therapy who presented after a ground-level fall with head trauma and an admission international normalized ratio (INR) of 9.0. Initial computed tomography (CT) of the head demonstrated only a scalp hematoma without intracranial hemorrhage, with the patient being neurologically intact. Approximately 12 hours later, worsening headache prompted repeat CT imaging, which revealed a new subarachnoid hemorrhage with intraventricular extension and an INR of 10.2 despite withholding warfarin since admission. Anticoagulation was promptly reversed with intravenous vitamin K and prothrombin complex concentrate (PCC), and serial imaging demonstrated a stable hemorrhage without the need for neurosurgical intervention. PCC was selected over fresh frozen plasma as it provides more rapid INR correction with a substantially lower infusion volume. Given the patient's high thromboembolic risk from a mechanical mitral valve, anticoagulation was cautiously resumed with heparin bridging followed by warfarin after hemorrhage stabilization. Recent ciprofloxacin exposure, acute alcohol intoxication, and marijuana use were identified as potential contributors to the markedly elevated INR. This case highlights the importance of repeat neuroimaging in anticoagulated patients with new or worsening neurologic symptoms despite initially negative imaging, as well as the challenges of balancing hemorrhagic and thromboembolic risks during anticoagulation reversal and reinitiation.

## Introduction

Warfarin remains the first-line anticoagulant for preventing thromboembolic events in patients with mechanical mitral heart valves. Although highly effective, warfarin has a narrow therapeutic window and numerous significant drug-drug interactions, making dosing difficult while also increasing the risk of bleeding complications [1]. Because mechanical valves in the mitral position carry a higher thromboembolic risk than those in the aortic position, a higher intensity of anticoagulation is required. Current guidelines recommend vitamin K antagonist therapy with a target INR of 2.5-3.5 [2]. Alternative agents, such as low-molecular-weight heparin, are primarily used for bridging therapy because warfarin remains the preferred agent for long-term thrombosis prevention in this population [3].

Intracranial hemorrhage is one of the most serious complications associated with anticoagulation. Delayed intracranial hemorrhage following an initially negative CT is uncommon, with a reported incidence of approximately 0.6% among patients receiving warfarin after blunt head trauma [4]. Despite its relatively low incidence, delayed intracranial hemorrhage remains clinically important because an initially negative computed tomography (CT) scan does not completely exclude subsequent hemorrhage development [4].

Management becomes even more complex in patients with mechanical mitral valves because interruption of anticoagulation increases the risk of thromboembolic events, whereas continuation or early reinitiation of anticoagulation may increase the risk of hemorrhagic expansion. This creates a delicate balance between preventing valve thrombosis and avoiding worsening intracranial bleeding.

It is also important to consider drug-drug and drug-substance interactions in patients presenting with elevated INR. Fluoroquinolones, such as ciprofloxacin, may potentiate warfarin by inhibiting cytochrome P450-mediated metabolism, particularly CYP1A2, thereby increasing anticoagulant effect and INR [5,6]. Several case reports have demonstrated clinically significant INR elevation in patients concurrently using warfarin and cannabis products, further supporting the potential clinical significance of this interaction [7,8]. Additionally, acute alcohol intake may inhibit warfarin metabolism and increase the anticoagulant effect [9].

This case report highlights delayed traumatic subarachnoid hemorrhage following initially negative CT imaging in a patient on chronic warfarin therapy for a mechanical mitral valve with a markedly supratherapeutic INR. The case was further complicated by multiple potential warfarin-potentiating exposures, including recent ciprofloxacin use, acute alcohol intoxication, and marijuana use. This case emphasizes the importance of repeat neuroimaging in anticoagulated patients with worsening symptoms, thorough medication reconciliation, and individualized anticoagulation management in patients with competing hemorrhagic and thromboembolic risks.

## Case presentation

A 76-year-old Caucasian male patient with a mechanical mitral valve replacement (1996) requiring chronic warfarin anticoagulation presented to the emergency department after a ground-level fall following a syncopal episode at home. His additional medical history included atrial fibrillation with a permanent pacemaker, heart failure with reduced ejection fraction (40-45%), hypertension, hyperlipidemia, and gout. The patient reported multiple syncopal episodes in the days preceding presentation. His cardiologist had recently advised him to hold warfarin for several days due to a reported supratherapeutic international normalized ratio (INR) of approximately 7 (reference range: 2.5-3.5 for mechanical mitral valve).

Home medications included allopurinol 100 mg daily, atorvastatin 40 mg daily, furosemide 40 mg daily, gemfibrozil 600 mg daily, metoprolol 50 mg daily, sacubitril/valsartan 24/26 mg twice daily, temazepam 15 mg as needed at bedtime, warfarin 5 mg daily, sildenafil 100 mg as needed, brimonidine 0.2% ophthalmic drops three times daily, latanoprost 0.005% ophthalmic drops daily, and pantoprazole 40 mg daily.

**Figure 1:**
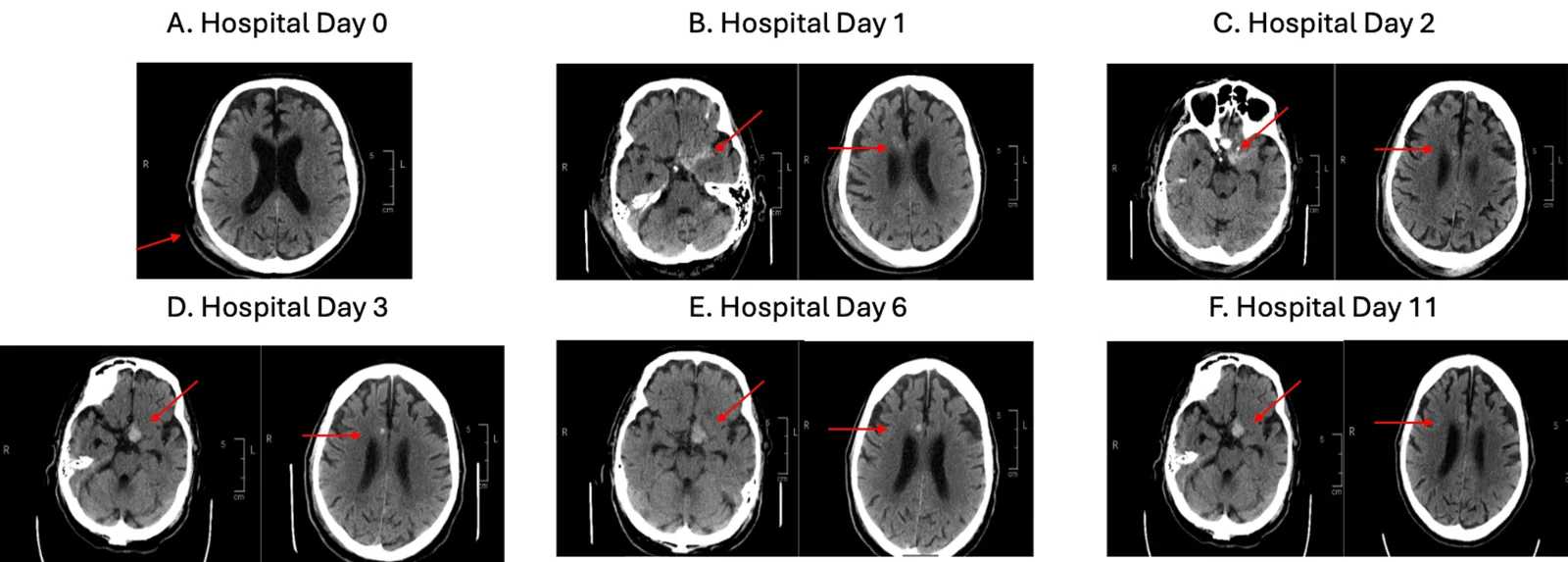
Serial noncontrast head CT imaging demonstrating the evolution of delayed intracranial hemorrhage. (A) Hospital day 0: Initial noncontrast CT head demonstrates a right parietal scalp hematoma (arrow) without acute intracranial hemorrhage. (B) Hospital day 1: Repeat CT demonstrates new acute subarachnoid hemorrhage involving the mesial right frontal lobe and posterior left temporal lobe with intraventricular extension into the lateral ventricle and a right frontal lobe contusion (arrows). (C) Hospital day 2: Persistent intracranial hemorrhage with overall stable appearance compared with the prior examination (arrows). (D) Hospital day 3: Persistent but improved in scattered subarachnoid hemorrhage (arrows). (E) Hospital day 6: Stable bilateral temporo-occipital subarachnoid hemorrhage and stable hemorrhage in the right anterior corpus callosum (arrows). (F) Hospital day 11: Decreased density of the subarachnoid hemorrhage and right anterior corpus callosum hemorrhage, consistent with interval improvement (arrow).

On presentation, the patient was hypotensive (99/57 mmHg) and was noted to have a large right parietal scalp hematoma. Laboratory evaluation demonstrated an INR of 9.0 (laboratory reference range: 0.8-1.2; therapeutic goal: 2.5-3.5 for a mechanical mitral valve) and hemoglobin of 12.9 g/dL (reference range: 13.5-17.5 g/dL). Blood alcohol level was elevated at 293 mg/dL (reference range: <10 mg/dL), consistent with severe intoxication, and urine drug screening was positive for marijuana (reference result: negative). Marijuana use was reported to be recreational in nature. Noncontrast computed tomography (CT) of the head demonstrated a right parietal-occipital scalp hematoma without acute intracranial findings. The patient's chronological clinical course is summarized in Table 1, and serial noncontrast head CT images demonstrating the evolution of the intracranial hemorrhage throughout hospitalization are shown in Figure 1. Osteopathic structural examination demonstrated increased suboccipital muscle hypertonicity, suboccipital tension, cervical fascial restriction, T1-T5 tissue texture abnormalities, and rib dysfunctions associated with chronic cardiopulmonary disease. Due to concern for evolving intracranial pathology and medical instability, osteopathic manipulative treatment was deferred.

**Table 1:** Chronological clinical course during hospitalization. Clinical events are summarized by hospital day. Laboratory reference range for INR: 0.8-1.2. Therapeutic INR goal for this patient with a mechanical mitral valve: 2.5-3.5. INR: international normalized ratio; PCC: prothrombin complex concentrate.

Hospital day	Clinical course
Day 0	Syncopal fall, hypotension (99/57), INR 9.0
Day 1	Severely worsened headache, INR 10.2; anticoagulation reversed with IV vitamin K and PCC
Day 2	INR 1.2; heparin infusion initiated
Day 3	Continued heparin infusion; BP 99/53; metoprolol decreased
Day 6	Warfarin restarted with bridge; last day of heparin drip
Day 11	Enoxaparin bridge; INR 1.9

Given the absence of intracranial hemorrhage and a normal neurologic examination, anticoagulation was held without immediate reversal, and the patient was admitted for observation. On the morning of hospital day 1, the patient reported significantly worsening headache. Repeat CT imaging obtained approximately 12 hours after the initial study demonstrated new intracranial hemorrhage with subarachnoid blood involving the cerebral hemispheres, particularly the mesial right frontal lobe and posterior left temporal lobe, with intraventricular extension into the dependent portions of both lateral ventricles. At that time, the INR had increased to 10.2.

Neurosurgery was consulted and recommended no acute neurosurgical intervention because the patient's neurologic status remained stable and serial imaging demonstrated no significant hemorrhage progression. Anticoagulation was reversed with intravenous vitamin K 5 mg and 1,920 units of prothrombin complex concentrate. The patient was transferred to the intensive care unit for close neurologic monitoring.

Serial CT imaging demonstrated stable to mildly improved hemorrhage throughout hospitalization. Following reversal, the INR decreased to 1.2 by hospital day 2. Given the patient's high thromboembolic risk secondary to a mechanical mitral valve, a low-dose heparin infusion was cautiously restarted on hospital day 2 following stable imaging studies. Warfarin therapy was reinitiated on hospital day 6 while bridging anticoagulation continued.

Hemoglobin progressively declined from 12.9 g/dL on admission to a nadir of 6.0 g/dL on hospital day 11. No overt source of additional bleeding was identified clinically, and serial neuroimaging demonstrated no significant expansion of the intracranial hemorrhage. The patient received one unit of packed red blood cells, with improvement in hemoglobin to 8.4 g/dL. Hemoglobin remained low but stable thereafter and was 8.8 g/dL at discharge.

Further medication reconciliation revealed recent use of a compounded budesonide/ciprofloxacin nasal preparation for sinus congestion. The medication was discontinued. The patient's markedly supratherapeutic INR was suspected to be multifactorial, with contributions from recent ciprofloxacin exposure, acute alcohol intoxication, and marijuana use.

The patient remained neurologically stable throughout the remainder of hospitalization. He was discharged home on hospital day 17 with warfarin 5 mg daily, enoxaparin 100 mg subcutaneously every 12 hours for bridging, a discharge INR of 2.2, and close outpatient cardiology follow-up.

## Discussion

Delayed intracranial hemorrhage

Delayed intracranial hemorrhage remains a recognized but diagnostically challenging complication of head trauma in anticoagulated patients. Prior studies have described delayed hemorrhage despite initially negative CT imaging, particularly in patients receiving chronic warfarin therapy with elevated INR values. This supports a standard of care regarding a low threshold for obtaining repeat neuroimaging when new or worsening neurologic symptoms develop despite initially reassuring imaging findings [4]. This patient's clinical course reinforces the importance of maintaining a high index of suspicion for delayed hemorrhage and close attention to changes in reported symptoms in anticoagulated patients, even when initial imaging is negative.

Anticoagulation management in mechanical mitral valve patients

Management of anticoagulation presents a significant clinical challenge, particularly in patients with mechanical mitral valves who are at high risk for thromboembolic events in the absence of adequate anticoagulation. Current guidelines continue to recommend lifelong anticoagulation with vitamin K antagonists, such as warfarin, for patients with mechanical heart valves [2]. Therefore, interruption of anticoagulation must be minimized while balancing the risk of active hemorrhage.

Because the patient initially presented with a supratherapeutic INR without evidence of intracranial hemorrhage, temporary discontinuation of warfarin without immediate reversal was initially appropriate. However, once repeat imaging demonstrated active intracranial bleeding and the INR increased to 10.2, prompt reversal of anticoagulation became necessary despite the inherent risk of valve thrombosis. Anticoagulation was subsequently reintroduced after radiographic stability was achieved using unfractionated heparin because of its short half-life and rapid reversibility before cautiously transitioning back to warfarin. This approach reflects the dynamic risk assessment required when both hemorrhagic and thromboembolic complications carry substantial morbidity and mortality. Previous studies evaluating anticoagulation-associated intracranial hemorrhage have similarly emphasized the challenge of balancing hemorrhage stabilization with prevention of thromboembolic complications when determining the timing of anticoagulation resumption [10].

Drug-drug and drug-substance interactions

Medication reconciliation and substance use assessment are essential components of evaluating patients who present with unexplained supratherapeutic anticoagulation. In this patient, the markedly elevated INR was likely multifactorial. Further investigation revealed recent use of a compounded budesonide/ciprofloxacin nasal preparation. Fluoroquinolones, including ciprofloxacin, have been associated with potentiation of warfarin's anticoagulant effect through inhibition of hepatic metabolism, resulting in elevated INR values and increased bleeding risk [5,6].

Acute alcohol intoxication likely represented an additional contributing factor. The patient's admission blood alcohol concentration of 293 mg/dL was consistent with severe intoxication [8]. Acute alcohol exposure may further inhibit warfarin metabolism and increase its anticoagulant effect [8]. Urine drug screening was also positive for marijuana use. Emerging evidence suggests that cannabinoids may potentiate warfarin activity through inhibition of CYP2C9, the primary enzyme responsible for warfarin metabolism [7]. A systematic review of published case reports found that warfarin dose reductions of 22-31% were often required to maintain therapeutic INR values in patients concurrently using cannabinoid products [7].

Although the relative contribution of each exposure cannot be determined, the coexistence of recent ciprofloxacin exposure, acute alcohol intoxication, and marijuana use likely contributed to this patient's markedly supratherapeutic INR and subsequent hemorrhagic complications.

Clinical practice implications

This case also highlights the importance of multidisciplinary collaboration in the management of medically complex patients. Coordination among internal medicine, neurology, neurosurgery, and cardiology facilitated individualized anticoagulation management while balancing competing hemorrhagic and thromboembolic risks. Although the patient was ultimately discharged in stable condition, this case illustrates the importance of repeat neuroimaging following new neurologic symptoms despite initially negative CT findings, careful anticoagulation reversal and reinitiation, and comprehensive evaluation of potential medication and substance interactions to optimize patient outcomes.

## Conclusions

Delayed intracranial hemorrhage should remain an important clinical consideration in anticoagulated patients following head trauma, even when initial CT imaging is negative. In patients with mechanical heart valves, management requires continuous reassessment of competing hemorrhagic and thromboembolic risks while guiding anticoagulation reversal and reinitiation. This case also emphasizes the importance of repeat neuroimaging in patients with evolving neurologic symptoms and careful evaluation of medication and substance interactions that may contribute to supratherapeutic anticoagulation.
